# Effect of Coenzyme Q_10_ on Physical Performance in Older Adults with Statin-Associated Asthenia: A Double-Blind, Randomized, Placebo-Controlled Clinical Trial

**DOI:** 10.3390/jcm13133741

**Published:** 2024-06-26

**Authors:** Federica Fogacci, Marina Giovannini, Giuliano Tocci, Egidio Imbalzano, Claudio Borghi, Arrigo F. G. Cicero

**Affiliations:** 1 Hypertension and Cardiovascular Risk Factors Research Centre, Medical and Surgical Sciences Department, Alma Mater Studiorum University of Bologna, 40138 Bologna, Italy; marina.giovannini3@unibo.it (M.G.); claudio.borghi@unibo.it (C.B.); arrigo.cicero@unibo.it (A.F.G.C.); 2 Cardiology Unit, Division of Cardiology, Department of Clinical and Molecular Medicine, Faculty of Medicine and Psychology, University of Rome ‘La Sapienza’, Sant’Andrea Hospital, 00198 Rome, Italy; giuliano.tocci@uniroma1.it; 3 Department of Clinical and Experimental Medicine, University of Messina, 98122 Messina, Italy; egidio.imbalzano@unime.it; 4 Cardiovascular Medicine Unit, IRCCS AOU BO, 40138 Bologna, Italy

**Keywords:** asthenia, coenzyme Q_10_, elderly, statin, physical performance, randomized clinical trial

## Abstract

**Background:** Available evidence from randomized clinical trials is contrasting and definitely inconclusive in determining whether or not CoQ_10_ dietary supplementation is advisable in patients with statin intolerance or poor statin tolerability. **Methods:** This randomized, double-blind, placebo-controlled clinical study aimed at investigating the effect of chronic dietary supplementation with coenzyme Q_10_ (CoQ_10_) phytosome on physical performance in older adults with a ≥3-month history of statin-associated asthenia. The study’s participants were randomized to either a placebo or 300 mg daily CoQ_10_ phytosome (equivalent to 60 mg CoQ_10_; Ubiqsome^®^, Indena SpA, Milan, Italy). Asthenia, handgrip strength (HGs), 2-min step test (2MST), and 1-min sit-to-stand (STS) repetitions were assessed at baseline and at 8-week follow-up. **Results**: After the first 4 weeks of dietary supplementation, individuals taking CoQ10 phytosome showed a greater improvement in asthenia compared to the placebo group (*p* < 0.05). Even more significantly, at 8-week follow-up, participants receiving CoQ10 showed substantial improvements in asthenia (−30.0 ± 20.0%), HGS (+29.8 ± 3.6%), 2MST (+11.1 ± 1.8%), and 1-min STS repetitions (+36.4 ± 3.9%) compared to both baseline and placebo (*p* < 0.05). **Conclusions**: According to our findings, chronic dietary supplementation with CoQ_10_ phytosome significantly enhances physical performance in older adults with statin-associated asthenia. This could have relevant implications for improving the compliance of older adults with statin treatment.

## 1. Introduction

Atherosclerotic cardiovascular diseases (ASCVD) are the leading cause of disability and death in developed countries [1].

Historically, Mendelian randomization studies have first shown that a lifetime reduction in low-density lipoprotein cholesterol (LDL-C) of ~40 mg/dL is able to reduce by over 50% the risk of developing ASCVD [2]. Afterward, randomized clinical studies have confirmed that reduced LDL-C correlates with CV risk decrease [3], so over time, lipid-lowering therapy (LLT) has become a cornerstone in CV risk reduction.

In accordance with the International guidelines, statins are the preferred initial LLT, being a cheap, widely available, safe, and extremely effective therapeutic tool [4]. Unfortunately, statin intolerance is an important cause of medication discontinuation [5,6]. It is largely recognized that the most common symptoms resulting in statin intolerance or statin discontinuation are statin-associated muscle symptoms (SAMS, including myalgia, cramps, and asthenia), which have also been strongly associated with increased risk of CV events [7].

SAMS pathogenesis is yet debated and a number of hypotheses have been formulated. Beyond a genetic background, SAMS could be induced by mitochondrial dysfunction mediated by 3-hydroxy-3-methylglutaryl coenzyme A reductase inhibition (and consequent inhibition of Coenzyme Q_10_ synthesis) and perturbation of post-translational protein prenylation [8].

A recent comprehensive meta-analysis of seven clinical studies (including 321 patients overall) failed to show that dietary supplementation with CoQ_10_ is either able to ameliorate statin-associated muscle pain or improve adherence to statin therapy [9]. However, the effect of CoQ_10_ supplementation on physical performance and statin-associated asthenia has never been investigated before. Furthermore, it should be considered that most of the studies included in the meta-analysis tested low-dose CoQ_10_ [9]. This is indeed a critical issue, given that the bioavailability of CoQ10 is generally low and varies significantly depending on the form of preparation—whether powder-filled capsules, tablets, or oil suspensions in soft gel capsules [10]. Recently, the development of phytosome technology has improved the bioavailability of CoQ_10_, increasing it by three times compared to standard pharmaceutical formulations and definitely improving its effectiveness in clinical practice [11,12]. Thus, the present study aimed to investigate the effect of chronic dietary supplementation with CoQ_10_ phytosome on physical performance in older adults with self-reported statin-associated asthenia.

## 2. Methods

### 2.1. Study Design and Participants

This was a randomized, double-blind, placebo-controlled clinical study aiming to assess the effects of dietary supplementation with CoQ_10_ phytosome on physical performance in older adults with self-reported statin-associated asthenia.

Participants were consecutively enrolled in the outpatient service of CV disease prevention of the Medical and Surgical Sciences Department of the University of Bologna, Bologna, Italy. Eligible patients were 65–80 years old individuals free from ASCVD, who had been on statin monotherapy (i.e., on the same statin at the same dosage) for at least 6 months and who had claimed statin-associated asthenia for at least 3 months at the study entry. Enrolled subjects did not have any serious or disabling diseases (e.g., severe organ failure, malignancy, or dementia), orthopedic disorders, myopathies, or any other acute or chronic disease that could significantly affect the individual’s perception of asthenia. These limiting criteria are needed to limit the interference of diseases, other drugs, and psychological-related disorders on self-perceived asthenia. Further exclusion criteria were obesity (body mass index (BMI) > 30 kg/m^2^) because it is not clear if BMI affects CoQ10 effectiveness and known gastrointestinal disorders potentially affecting the absorption of CoQ_10_.

**Figure 1:**
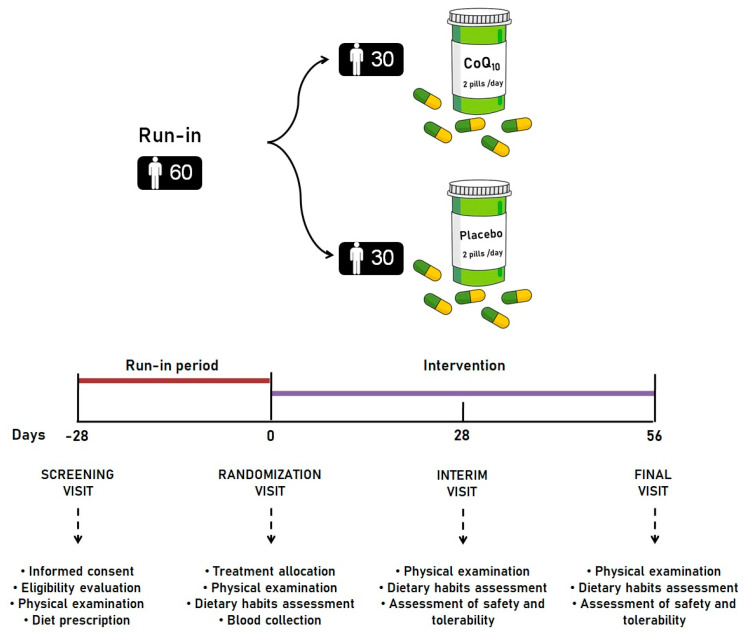
Design and timeline of the study. All pictures shown are for illustration purposes only since the study was designed as double-blind.

As per our clinical practice, enrolled subjects adhered to an overall healthy lifestyle, including a Mediterranean diet for four weeks before being randomized to receive dietary supplementation with CoQ_10_ phytosome or placebo. The adherence to the Mediterranean diet pattern has been monitored by the use of a validated semi-quantitative questionnaire [13]. The intervention period lasted 8 weeks. At baseline, the patients were evaluated for clinical status, and by the execution of a physical examination and laboratory analyses. During the study, the patients were required not to change their standard physical activity. The physical examination was repeated after 4 and 8 weeks of intervention. The timeline of the study has been reported in detail in Figure 1.

The study fully was conducted in full compliance with the ethical guidelines of the Declaration of Helsinki and with The International Council for Harmonisation of Technical Requirements for Registration of Pharmaceuticals for Human Use (ICH) Harmonized Tripartite Guideline for Good Clinical Practice (GCP). The study protocol received approval from the Ethical Committee of the University of Bologna (Internal Code: QELD_Stat_2017) and registered in ClinicalTrials.gov (ID: NCT06391606). All patients signed a written informed consent to participate.

### 2.2. Treatment

After a 4-week period of diet and lifestyle standardization, as per our clinical practice, study participants were randomized to receive daily supplementation either with 2 indistinguishable pills of placebo or 150 mg CoQ_10_ phytosome (i.e., 300 mg CoQ_10_ phytosome per day, equivalent to 60 mg CoQ_10_; Ubiqsome^®^, kindly provided by Indena SpA, Milan, Italy), with same color, shape, dimension, and taste/aftertaste.

Randomization was centrally performed, by computer-generated codes. Participants and investigators were blinded to the group assignment. The investigators sequentially attributed a numbered box including 60 pills to each enrolled subject following the randomization list. Randomization codes were kept in a sealed envelope that was opened after study completion and data analysis.

At the end of the clinical trial, all unused pills were retrieved for inventory. Treatment compliance was assessed by counting the number of returned pills. Mean compliance was calculated by the percentage ratio of the number of returned pills to the number of pills expected to have been consumed based on the number of days of active treatment during the study.

### 2.3. Assessments

#### 2.3.1. Clinical Data and Anthropometric Measurements

Information collected from the patients’ histories included the presence of ASCVD and other systemic diseases, as well as allergies and current medications. Validated semi-quantitative questionnaires, including the Food Frequency Questionnaire (FFQ) [14], were utilized to assess demographic variables, recreational physical activity, dietary habits, and smoking habits. Self-reported asthenia was assessed on a 10-point visual analog scale (VAS), considering 0 = absence of asthenia and 10 = extreme asthenia.

Waist circumference (WC) was measured in a horizontal plane at the end of a normal expiration, at the midpoint between the lower margin of the last rib and the top of the iliac crest. Height and weight were measured to the nearest 0.1 cm and 0.1 kg, respectively, with individuals standing erect, eyes facing forward, wearing light clothing and bare feet. Body Mass Index (BMI) was calculated by dividing body weight in kilograms by height in meters squared (kg/m^2^).

#### 2.3.2. Handgrip Strength Test

A hand-held dynamometer was used to assess handgrip strength (HGS), which is widely recognized as a surrogate measure of whole-body strength [15]. According to international standard protocols, individuals were asked to comfortably sit with the elbow near the trunk, bent at 90°, and the hand in a neutral position with the thumb pointing upwards. The measure was obtained after participants performed one familiarization trial with both hands, and the highest reading out of three (in kg) was used as the study variable [16].

#### 2.3.3. Dynamic Physical Performance

Muscular strength of the lower limbs was assessed by the 1-min sit-to-stand (STS) test [17]. According to standardized protocols, the study’s participants were asked to sit and stand up from their chair repeatedly, as quickly as possible, over a 1-min course. The STS test started following verbal commands, and patients were notified when 15 s remained. The number of repetitions was counted and used as the study variable.

Functional aerobic endurance and functional fitness were evaluated by the 2-min step test (2MST) [18]. The test required that individuals step in place as fast as possible for 2 min while lifting the knees to a height midway between their patella and iliac crest. Performance on the test was defined as the number of right-side steps of the criterion height, as completed in 2 min.

#### 2.3.4. Laboratory Analyses

Biochemical analyses were carried out on venous blood withdrawn after overnight fasting (at least 12 h). Serum was obtained by the addition of disodium ethylenediaminetetraacetate (Na2EDTA) (1 mg/mL) and blood centrifugation at 3000 RPM for 15 min at 25 °C.

Trained personnel performed laboratory analyses immediately after centrifugation, according to standardized methods [19]. The following parameters were directly assessed: total cholesterol (TC), high-density lipoprotein cholesterol (HDL-C), triglycerides (TG), fasting plasma glucose (FPG), alanine transaminase (ALT), and aspartate transaminase (AST), gamma-glutamyl transferase (GGT) and CPK.

LDL-C was obtained by the Friedewald formula [20]. Non-HDL cholesterol (Non-HDL-C) resulted from the difference between TC and HDL-C. The glomerular filtration rate (eGFR) was estimated by the Chronic Kidney Disease Epidemiology Collaboration (CKD-epi) equation [21].

#### 2.3.5. Blood Pressure Measurements

Blood pressure (BP) was measured following the recommendations of the International Guidelines for the management of arterial hypertension [22]. Resting systolic (SBP) and diastolic BP (DBP) were measured using a validated oscillometric device with an appropriately sized cuff placed on the right upper arm. To enhance detection accuracy, three BP readings were taken at 1-min intervals. The first reading was discarded, and the average between the second and the third readings was recorded as the study variable.

#### 2.3.6. Assessment of Safety and Tolerability

Safety and tolerability were assessed through continuous monitoring continuous monitoring throughout the study, which included detecting any adverse events, clinical safety evaluations, laboratory findings (i.e., liver enzymes, CPK), vital sign measurements (blood pressure, heart rate), and physical examinations. An independent expert clinical event committee, blinded to the study, was appointed by the principal investigator (P.I.) to classify any adverse events that occurred during the trial. These events were categorized as not related, unlikely related, possibly related, probably related, or definitely related to the investigated product [23].

### 2.4. Statistical Analysis

Data were analyzed using intention to treat by means of the Statistical Package for Social Sciences (SPSS) version 25.0 (IBM Corporation, Armonk, NY, USA) for Windows.

No previous study specifically addressed the effect of CoQ_10_ supplementation on physical performance in older adults with statin-related asthenia. Consequently, based on the possible effects of different dietary supplements on muscular strength in older adults [24,25], we estimated that the sample size needed to detect a 5% between-group mean difference in HS could be 27 subjects per group, assuming a power of 0.90 and an alpha error of 0.05. Considering the risk of non-compliance to diet and/or treatment and of withdrawal from the study, we enrolled 30 patients per group. As per protocol, we decided a priori to check the efficacy of treatments in subjects assuming at least 90% of the investigational product doses foreseen by the trial design.

The normality distribution of the studied variables has been tested by the use of the Kolmogorov–Smirnov test. Efficacy analyses were conducted on the intention-to-treat (ITT) population, which included all subjects with at least one post-baseline control. Additionally, a sensitivity analysis of the primary variable was planned for the per-protocol population (PPP). A comprehensive descriptive analysis of the collected parameters was performed. Categorical variables were presented as absolute numbers and percentages and compared using either Fisher’s exact test or the Wilcoxon rank–sum test, depending on whether they were nominal or ordinal. Continuous variables were expressed as mean ± standard deviation (SD) and compared by analysis of variance (ANOVA) followed by a post hoc Tukey test as they were all normally distributed in the sample. Then, a repeated-measure ANOVA (with time as a within-subject factor and intervention group as a between-subject factor) was carried out.

The minimum level of statistical significance was set to *p* < 0.05 for two-tailed tests. Dixon’s Q test was consistently applied to exclude the extreme values.

## 3. Results

**Figure 2:**
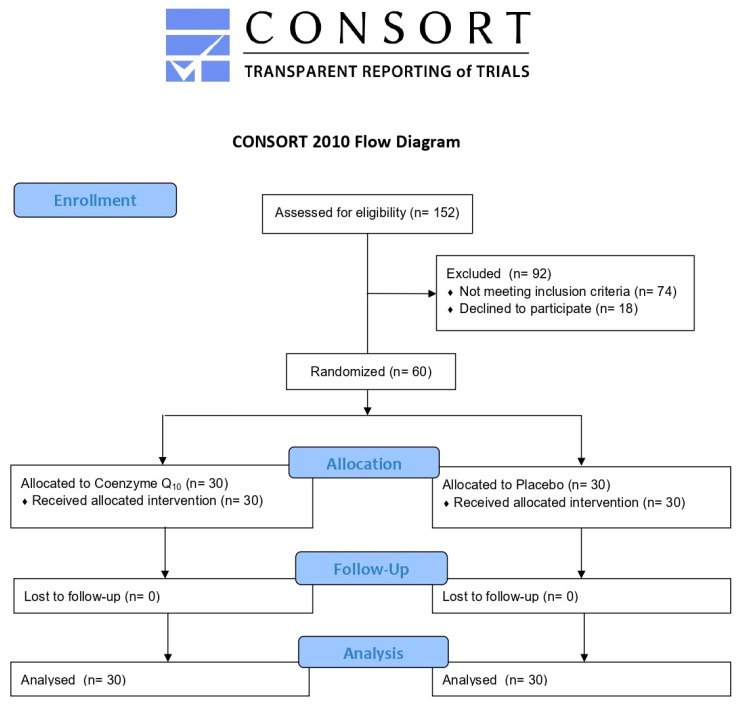
CONSORT flow diagram of the clinical study.

A total of 152 volunteers were consecutively assessed for eligibility. Sixty volunteers entered the run-in period and were randomized to take active treatment and placebo. All enrolled subjects successfully completed the study according to the trial protocol (Figure 2).

All enrolled individuals (Men: 33, Women: 27) completed the clinical trial according to the study design (dropout rate = 0%), and no protocol violations were reported. The enrolled subjects declared not to have changed their standard physical activity intensity or frequency during the trial.

Compliance with treatment was 100% both in the active treated group and in the placebo group.

At baseline, the study groups were well matched for all relevant clinical and demographic data, without significant differences between the distributions of the sampled parameters (Table 1). Six patients in the Coenzyme Q10 treated group and five in the placebo-treated one declared to be affected by unspecific forms of artromyalgia (migrant, asymmetric, with variable intensity). 

After both the first 4 and 8 weeks of treatment, no change has been observed as regards anthropometric and hematochemistry parameters in both groups of treatment (*p* always >0.05). After the first 4 weeks of treatment, both groups experienced significant improvement in asthenia compared to baseline. The between-group difference was also statistically significant (Table 2).

Four more weeks later (week 8), the effect on asthenia was sustained –versus placebo and baseline- only in the actively treated group. Statistical significance was reached both versus baseline and versus placebo (Table 2). A significant time*group interaction in VAS was also observed (F = 9.235, *p* = 0.009). At 8-week follow-up, CoQ10 phytosome was associated with significant improvements in HGs, 1-min STS repetitions and 2MST, as compared to baseline and placebo (confirmatory secondary endpoints; Table 2). Significant time*group interactions for HGs (F = 16.321, *p* = 0.012), 1-min STS repetitions (F = 9.762, *p* = 0.027) and 2MST (F = 9.035, *p* = 0.011) were observed, as well.

A significant improvement of SBP vs. baseline was also observed in the CoQ10 phytosome-treated group at week 8 (*p* = 0.031). 

At the end of the study, one of the six patients claiming myalgia before the study reported the disappearance of the symptoms. 

## 4. Discussion

In our double-blind, randomized, placebo-controlled clinical trial, middle-term dietary supplementation with CoQ_10_ phytosome was effective in improving physical performance measured by different tests in older adults with statin-associated asthenia. In particular, in the CoQ_10_-treated patients, VAS for asthenia decreased by 30.0 ± 20.0%, HGc increased by 29.8 ± 3.6%, 1-min STS repetitions by 36.4 ± 3.9%, and 2MST by 11.1 ± 1.8% (*p* < 0.05 vs. baseline and vs. placebo). The observed results were enhanced by the use of a specific phytosome delivery formulation (patented as Ubiqsome^®^) that has already been previously shown to improve the oral absorption of coenzyme CoQ_10_ and optimize the physiological plasma levels of CoQ_10_ after just a single dose [26,27]. 

Statins are among the most commonly used drugs all around the world. While statin efficacy in terms of cardiovascular disease reduction has been confirmed in older adults [28], the lack of compliance with the treatment is associated with an increase in the risk itself [29]. In particular, in a large cohort of older adults, the ones discontinuing statin treatment experienced significantly higher risk of hospital admissions for heart failure (Hazard Ratio [HR] 1.24, 95% Confidence Interval [CI] 1.07–1.43) and any cardiovascular outcome (HR 1.14, 95%CI 1.03–1.26), and deaths from any cause (HR 1.15, 95%CI 1.02–1.30), compared with patients continuing statin treatment [29].

Statin treatment has been previously shown to decrease the circulating levels of CoQ_10_, and histopathological findings support the pivotal role of mitochondrial dysfunction in the pathogenesis of SAMS [30]. The reason why circulating CoQ_10_ is reduced in patients undergoing statin treatment has not yet been clarified. However, the most well-corroborated hypotheses support that statin treatment may either decrease the biosynthesis of CoQ_10_ through inhibition of the mevalonate pathways [31] or decrease the absorption of dietary CoQ_10_ through modulation of microbiota [32]. If these hypotheses are confirmed through rigorous research, dietary supplementation with CoQ_10_ would be advisable in patients on statins not specifically in order to counteract SAMS but rather to maintain adequate levels of CoQ_10_ for the realization of physiological functions and physical activity.

Nevertheless, available evidence from randomized clinical trials is contrasting and definitely inconclusive in determining whether or not CoQ_10_ dietary supplementation is advisable in patients with statin intolerance or poor statin tolerability [33]. Indeed, previous randomized clinical studies were carried out by testing low-dose CoQ_10_ and led to conflicting—though generally negative—results, in particular on myalgia [34]. More recently, Chen et al. [35] reported in a retrospective study that CoQ_10_ users experienced a similar SAMS resolution frequency than non-Q_10_ users (25% vs. 31%, respectively; unadjusted odds ratio [OR] 0.75, 95%CI 0.41–1.38; *p* = 0.357), even after adjustment for SAMS risk factors (OR 0.84, 95%CI 0.45–1.55, *p* = 0.568) or for significant differences among CoQ_10_ users and non-users (OR 0.82, 95%CI 0.45–1.51, *p* = 0.522). However, considering the clinical heterogeneity of the considered sample, the low number of CoQ_10_ treated subjects, the different CoQ_10_ doses and formulations used by the patients, as well as the incomplete information on the timing and duration of the CoQ_10_ intake, it is hard to conclude for a non-effect of CoQ_10_ on SAMS based on these data. The study of Dohlmann et al. concluded that 400 mg supplementation with CoQ_10_ had no effect on statin-related myalgias. This small trial was, however, powered on changes in plasma CoQ_10_ and mitochondrial measurements and not on clinical symptoms [36]. To the best of our knowledge, our clinical trial is the first one focusing on the effect of supplementation with a high-bioavailability CoQ_10_ formulation on statin-related asthenia, in spite of myalgia. The evaluation of the effect of CoQ10 on a symptom that could appear before myalgia, in particular in subjects with less muscular mass, could be of particular interest considering that the antioxidant and anti-inflammatory properties of CoQ10, as well as its ability to improve the bioenergetics of the muscle cells, may be more useful when the muscle health is not frankly compromised, as clearly demonstrated in healthy athletes [37].

On the other side, CoQ_10_ is a natural antioxidant compound that offers potential benefits in the management of patients affected by CVD, preventing the damage induced by free radicals and the activation of inflammatory signaling pathways with pleiotropic effects [10,11]. In particular, CoQ_10_ could protect LDL from oxidation, improving LDL composition and endothelial function. Moreover, dietary supplementation with CoQ_10_ is safe, without any known pharmacological interactions [10,11]. The risk–benefit of CoQ_10_ supplementation has been recently confirmed in a huge systematic review and meta-analysis of 884 randomized controlled intervention trials of 27 micronutrients, including 883,627 participants (4,895,544 person-years), concluding that CoQ_10_ supplementation is one of the few able to significantly reduce all-cause mortality risk (relative risk [RR] 0.68, 95%CI 0.49 to 0.94) [38]. In our trial, even if relatively small and short-term, we also did not observe any CoQ_10_-associated adverse events. 

### Limitations

Despite the significant findings and potential practical implications, this study has certain limitations. Notably, the relatively short follow-up period does not provide insight into the possible occurrence of adaptation phenomena, although such phenomena have never been documented for CoQ10. Even if adequately powered for the primary outcome, the sample size of the study was relatively small. Then, statin-associated asthenia was self-reported, so potentially influenced by comorbidities, other drugs, or psychological factors, even if we enrolled overall healthy older adults. Of course, the selection of overall healthy subjects reduces the possibility of inference our results to more ill and polypharmacologically treated patients. On the other side, physical performances were assessed with validated tests. Furthermore, more research is needed that confirms our observations in the long term by directly comparing different CoQ_10_ pharmaceutical formulations. 

## 5. Conclusions

In conclusion, middle-term dietary supplementation with CoQ_10_ phytosome effectively improved physical performance in older adults with statin-associated asthenia. It could have relevant implications to improve the compliance of older adults to statin-treatment.

## Figures and Tables

**Table 1:** Main characteristics of the enrolled individuals at baseline (week 0).

Parameters	Overall Sample (Mean ± SD; *N.* 60)	Coenzyme Q_10_ (Mean ± SD; *N.* 30)	Placebo (Mean ± SD; *N.* 30)	*p*-Value (between Groups)
Age (years)	74 ± 3	74 ± 2	73 ± 3	0.13
Weight (kg)	72 ± 5	71 ± 4	73 ± 5	0.09
Waist circumference (cm)	92 ± 6	91 ± 5	93 ± 6	0.17
Body Mass Index (kg/m^2^)	25 ± 1	24 ± 1	25 ± 1	0.09
Systolic BP (mmHg)	136 ± 6	136 ± 5	135 ± 6	0.49
Diastolic BP (mmHg)	88 ± 3	87 ± 2	88 ± 3	0.13
TC (mg/dL)	201 ± 12	203 ± 11	199 ± 9	0.13
HDL-C (mg/dL)	46 ± 3	45 ± 3	47 ± 3	0.06
Non HDL-C (mg/dL)	158 ± 9	159 ± 9	157 ± 8	0.37
LDL-C (mg/dL)	115 ± 7	116 ± 6	113 ± 7	0.08
Triglycerides (mg/dL)	213 ± 17	216 ± 16	210 ± 14	0.13
FPG (mg/dL)	89 ± 4	88 ± 3	89 ± 4	0.28
AST (U/L)	24 ± 2	24 ± 3	23 ± 2	0.13
ALT (U/L)	25 ± 3	24 ± 2	25 ± 3	0.13
Gamma-GT (mg/dL)	36 ± 5	35 ± 3	36 ± 5	0.36
CPK (U/L)	164 ± 22	164 ± 19	163 ± 21	0.85
eGFR (mL/min)	73 ± 6	73 ± 5	72 ± 6	0.49

ALT = Alanine aminotransferase; AST = Aspartate aminotransferase; BP = Bood pressure; CPK = Creatine phosphokinase; eGFR = Estimated glomerular filtration rate; FPG = Fasting plasma glucose; Gamma-GT = Gamma glutamyl transferase; HDL-C = High-density lipoprotein cholesterol; LDL-C = Low-density lipoprotein cholesterol; N = Number of individuals; SD = Standard deviation; TC = Total cholesterol.

**Table 2:** Main characteristics of the enrolled individuals at baseline (week 0), 4-week and 8-week follow-up.

Parameters	Coenzyme Q_10_ (*N.* 30)			Placebo (*N.* 30)		
	Baseline	4-Week Follow-Up	8-Week Follow-Up	Baseline	4-Week Follow-Up	8-Week Follow-Up
Systolic BP (mmHg)	136 ± 5	134 ± 6	132 ± 5 *	135 ± 6	134 ± 5	133 ± 6
Diastolic BP (mmHg)	87 ± 2	86 ± 2	85 ± 3	88 ± 3	86 ± 2	86 ± 1
HGs (kg)	15.1 ± 0.8	16.5 ± 0.6	19.6 ± 0.5 *°^§^	15.2 ± 0.6	14.7 ± 0.7	15.0 ± 0.6
1-min STS repetitions	18.7 ± 2.9	21.5 ± 2.8	25.5 ± 2.7 *°^§^	18.4 ± 2.7	19.1 ± 2.8	18.5 ± 2.57
2MST (steps)	100.3 ± 5.9	105.5 ± 6.6	111.5 ± 5.5 *^§^	101.3 ± 6.4	100.8 ± 5.5	102.6 ± 5.8
VAS for asthenia	6 (3–8)	4 (2–6) *°	3 (2–5) *°^§^	5 (3–8)	4 (3–7) *	6 (3–8)

* *p* < 0.05 versus baseline; ° *p* < 0.05 versus placebo; ^§^ *p* < 0.05 for time*group interaction. 2MST = 2-min step test; BMI = Body mass index; HGs = Handgrip strength; Min = Minute; N = Number of individuals; STS = Sit-to-stand; VAS = Visual analogue scale.

## Data Availability

Data supporting the findings of this analysis are available from the Authors with the permission of the University of Bologna. The results from the present study have been presented during the 32nd European Meeting On Hypertension and Cardiovascular Protection (Milan, June 2023).
